# Studies on Preparation, Characterization and Application of Porous Functionalized Glycidyl Methacrylate-Based Microspheres

**DOI:** 10.3390/ma14061438

**Published:** 2021-03-16

**Authors:** Przemysław Pączkowski, Barbara Gawdzik

**Affiliations:** Department of Polymer Chemistry, Institute of Chemical Sciences, Faculty of Chemistry, Maria Curie-Sklodowska University in Lublin, Gliniana 33, 20-614 Lublin, Poland; barbara.gawdzik@umcs.pl

**Keywords:** seed swelling polymerization, polymeric microspheres, porous structure, surface modification, glycidyl methacrylate, functionalized material, green liquid chromatography

## Abstract

A one-step swelling and polymerization technique was used in the synthesis of porous glycidyl methacrylate (GMA) and ethylene glycol dimethacrylate (EGDMA) monodisperse polymeric microspheres. The polystyrene (PS) seed obtained in the dispersion polymerization was used as a shape template. The presence of epoxide rings in the chemical structure of microspheres enables their post-polymerization chemical modifications involving: the Diels-Alder reaction with sodium cyclopentadienide and maleic anhydride, the reaction with 4,4′-(bismaleimido)diphenylmethane, and the thiol-Michael reaction with methacryloyl chloride and 2-mercaptopropionic acid. Changing the reaction mixture composition—the amounts of crosslinking monomer and PS seed as well as the type and concentration of porogen porous microspheres of different porous structures were obtained. Their porous structures were characterized in the dry and swollen states. The copolymers obtained from the equimolar monomers mixture modified in the above way were applied as the column packing materials and tested in the reverse-phase HPLC (High-Performance Liquid Chromatography). A few factors influencing morphology and porous structure of microspheres were studied.

## 1. Introduction

Over years the synthesis of functional polymeric materials has drawn increasing attention due to their properties and perspective applications such as the support in the solid-phase synthesis of peptides or organic compounds and solid-phase catalysis, enzyme immobilization, and ion-exchange resin [1,2]. Moreover, they are used as sorbents in gas and liquid chromatography or solid-phase extraction [3,4].

Currently, out of concern for the natural environment and the safety and hygiene of chromatographers, but above all due to economic considerations, scientists are making attempts to apply green chromatography. You can become more “green” by using stationary phases from natural materials [5,6,7,8,9]. The next strategy for greening liquid chromatography is to search for green components for the mobile phase [10]. Typically, the solvents used in RPLC (Reverse Phase Liquid Chromatography) include a mixture of water with acetonitrile or methanol. Both of them are toxic, but the latter proves to be less toxic. The other green strategies in LC are based on the reduction of the amount of solvents used as mobile phase [11,12].

Among stationary phases for HPLC, an important role is played by polymeric sorbents, the chemical nature of which can be modified depending on the needs. The introduction of functional groups into the structure of non-polar styrene-divinylbenzene (St-DVB) copolymer can change its selectivity completely [13,14].

Each chemical modification also affects the porous structure of the sorbent. Therefore, it is important to get to know the structure of the sorbent.

To analyze its porous structure, direct and indirect techniques were used [15]. Microscopy (SEM, AFM) or X-ray analysis, as direct techniques, only provide information about the surface shape based on the surface imaging. The information about the surface area and pore volume could be also obtained. In addition to the imaging techniques for a more quantitative analysis an indirect one should be used. Gas sorption is the most common, because it makes it possible to understand the processes occurring in the pore volume and on the pore surface. Gas molecule sorption is determined by the pressure. This technique is not appropriate for measuring the extent of the flow through pores.

Inverse size-exclusion chromatography (ISEC), as a method based on the correlation between the molecules’ diffusivity into the pores with an appropriate pore diameter, could be a powerful tool for determining the pore size distribution of stationary phases in a wet, swollen state.

Polymeric microspheres derived from glycidyl methacrylate (GMA) are very attractive as a useful material with a broad range of applications. Copolymers are widely used as ion exchangers [16,17,18,19,20], macromolecular supports for enzyme immobilization [21,22,23] or catalysts [24], column packings for HPLC [10,11,12,25], and dye remover [26,27]. Microspheres based on GMA are also widely applied in bioseparation [28,29], cell isolation [30], nucleic acid and protein purification and immunoassay [31,32,33,34]. Fluorescence and encoded GMA-based microspheres can be used as fluorescent sensor [35] and material that shows a particularly important role in labeling, detection, and high-throughput screening [36,37].

This paper discusses the use of a new polymer material containing different polar groups on the surface as a stationary phase for the chromatographic column. For their preparation, one-step swelling polymerization of glycidyl methacrylate and ethylene glycol dimethacrylate (EGDMA) and their further functionalization were carried out. In particular, polymers with carboxylic groups which, due to the reduction of the amount of solvents used in HPLC, are part of the global trend of green chromatography strategies.

Additionally, information regarding the correlation between a few factors of synthesis conditions and morphology, as well as the porous structure, is provided. As a standard method for the surface area and porosity analyses nitrogen physisorption was used. For the functional stationary phases: epoxide, diol, and (di)carboxyl, an inverse size-exclusion chromatography as an alternative method was taken into account.

## 2. Experimental

### 2.1. Materials

In the synthesis and purification of polymeric microspheres, the following chemicals are used: styrene ≥99% (Fluka, Buchs, Switzerland), glycidyl methacrylate 99.5%, GMA (Sigma-Aldrich, St. Louis, MO, USA), ethylene glycol dimethylacrylate 98%, EGDMA (Sigma-Aldrich, St. Louis, MO, USA), 2,2′-azobisisobutyronitrile, AIBN (Fluka, Buchs, Switzerland), ethyl alcohol anhydrous 99.8% pure p.a. (POCh, Gliwice, Poland), polyvinylpyrrolidone, PVP-40 (Fluka, Buchs, Switzerland), sodium dodecyl sulfate ≥99%, SDS (Sigma-Aldrich, St. Louis, MO, USA) methanol 99.8% pure p.a. (POCh, Gliwice, Poland), tetrahydrofuran 99.5% pure (POCh, Gliwice, Poland), toluene 99.9% pure (Sigma-Aldrich, St. Louis, MO, USA), hexane 99.5% pure p.a. (POCh, Gliwice, Poland), dichloromethane 99.5% pure, (POCh, Gliwice, Poland), acetone 99.5% pure p.a. (POCh, Gliwice, Poland), hydrochloric acid 35–38% pure p.a. (POCh, Gliwice, Poland), sulfuric acid 30% pure p. a. (POCh, Gliwice, Poland), ammonium chloride (POCh, Gliwice, Poland).

In the functionalization of polymeric microspheres, the following chemicals are used: sodium cyclopentadienide, NaCp, 2 M in THF (Sigma-Aldrich, St. Louis, MO, USA), maleic anhydride 99% (Sigma-Aldrich, St. Louis, MO, USA), 4,4′-bis(maleimido)diphenylmethane 95%, BMI (Sigma-Aldrich, St. Louis, MO, USA), methacryloyl chloride 97% (Sigma-Aldrich, St. Louis, MO, USA), 2-mercaptopropionic acid 96%, 2-MPA (Sigma-Aldrich, St. Louis, MO, USA), trimethylamine anhydrous (POCh, Gliwice, Poland).

Mixtures of alkylbenzenes (toluene, ethylbenzene, propylbenzene, butylbenzene, pentylbenzene), alkyl aryl ketones (1-phenylethan-1-one, 1-phenylpropan-1-one, 1-phenylbutan-1-one, 1-phenylpentan-1-one, 1-phenylhexan-1-one, 1-phenylheptan-1-one), alkyl benzoates (methyl benzoate, ethyl benzoate, propyl benzoate, butyl benzoate, pentyl benzoate), *N*-alkylanilines (aniline, *N*-methylaniline, *N*-ethylaniline, *N*-propylaniline, *N*-butylaniline, *N*-pentylaniline) and alkyl aryl ethers (methoxybenzene, ethoxybenzene, propoxybenzene, butoxybenzene) obtained from the Department of Polymer Chemistry (Lublin, Poland) were used in the chromatographic studies.

For determination of the retention time of toluene, alkylphenones (1-phenylethan-1-one, 1-phenylbutan-1-one), phthalates (dimethyl phthalate, diethyl phthalate, dipropyl phthalate, dibutyl phthalate, dioctyl phthalate, dinonyl phthalate, didodecyl phthalate), and polystyrene standards with various molecular weights (580; 1050; 2450; 5100; 11,600; 30,300; 68,000; 120,000; 330,000; 750,000; 1,260,000; 2,750,000) from E. Merck (FRG, Darmstadt, Germany) were applied in the porous structure investigations by inverse size exclusion chromatography.

The solvents acetonitrile 99.9% (Merck, Darmstadt, Germany), methanol 99.8% (Merck, Darmstadt, Germany), and tetrahydrofuran 99.8% (Merck, Darmstadt, Germany) were used as mobile phases in HPLC and ISEC.

### 2.2. Dispersion Polymerization of PS Seed

Amounts of 1.25 g polyvinylpyrrolidone and 64 mL ethyl alcohol were placed in the 100 mL three-necked round-bottom flask equipped with a mechanical stirrer and a thermometer. The mixture of monomer (styrene, 16 mL) and initiator (AIBN, 0.58 g) was added into the non-aqueous solution while the stabilizer was dissolved. After purging the solution with a nitrogen gas, the round-bottom flask was immersed in a thermostated water bath with a rotation speed of 100 rpm at 70 °C for 8 h. The obtained spheres were centrifuged for 10 min at 6000 rpm while the supernatant was removed. Then the resulting material was washed with hot water and methanol to get rid of residual unreacted monomers and then re-isolated using a centrifuge. The purified polymer was then placed in an oven at 100 °C.

### 2.3. One-Step Swelling and Polymerization of Poly(GMA-co-EGDMA) Microspheres

One-stage swelling and polymerization was also conducted in a three-necked round-bottom flask equipped with a mechanical stirrer and a nitrogen gas inlet system.

In the first step, a 200 mL solution of 1% PVP and 0.25% SDS was prepared. The dried PS seeds (0.45 g) were redispersed into a 25 mL beaker containing 15 mL of PVP/SDS solution. The vessel was placed in the bath and the ultrasound was applied for about 1 min to “break up” any agglomerates.

A mixture of monomers (GMA and EGDMA, 13.4 mL), initiator (AIBN, 0.14 g) and porogenic solvent was prepared in a 50 mL beaker. The oil phase thus obtained was transferred to a 250 mL Erlenmeyer flask containing 150 mL of PVP/SDS solution. The whole was subjected to the homogenizer for about 5 min and then transferred to a 250 mL three-necked flask equipped with a mechanical stirrer and a thermometer. The solution was deoxygenated by bubbling the inert gas (nitrogen) through it for about 30 min. Then the rotation speed was set to 150 rpm.

The suspension of PS seeds was dropped into the flask using a plastic Pasteur pipette. The swelling process ran for 24 h at the temperature of 30 °C. The temperature was then increased to 70 °C to initiate the polymerization reaction and the process continued for another 24 h.

The resulting GE microspheres were filtered at the reduced pressure in a Büchner funnel. To rinse the surfactant and residual unreacted monomers from the system, the material was washed on the filter with large amounts of hot water, 20 mL of tetrahydrofuran, and 20 mL of acetone.

Due to the proposed use of polymer microspheres as the stationary phases in liquid chromatography, the polystyrene present in the material should be removed. The non-crosslinked linear polymer is soluble in organic samples and may distort the results of the chromatographic separation. For this purpose, the obtained GE microspheres were placed in a 250 mL single neck round bottom flask containing 100 mL of tetrahydrofuran. The polystyrene seeds are dissolved in this solvent which is also used as a mobile phase in chromatography. A flask equipped with a water jacketed reflux condenser was placed on a heating mantle. After about 4 h the microspheres were filtered in vacuum in a Büchner funnel and washed through the filter with pure tetrahydrofuran.

After deaerating with nitrogen, the polymerization was carried out at 70 °C for 24 h. The particles were dried in vacuum at ambient temperature.

### 2.4. Surface Modification

**Scheme 1:**
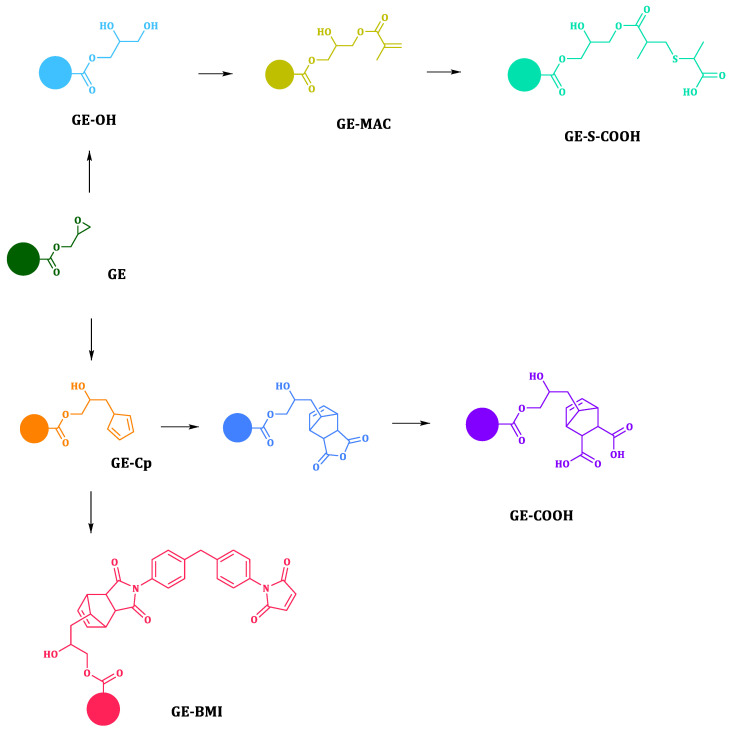
General routes of functionalization of GE polymeric microspheres.

Scheme 1 presents the routes of functionalization of GE polymeric microspheres. The glycidyl-based microspheres with carboxylic groups were synthesized by the Diels-Alder reaction with sodium cyclopentadienide and maleic anhydride in accordance with the procedure described earlier [38].

#### 2.4.1. Microspheres GE-Cp

Poly(GMA-co-EGDMA) microspheres (4 g, epoxide value, EV = 1.8 mmol g^−1^, 7.2 mmol of epoxide groups) were suspended in 100 mL of dry tetrahydrofuran in a round bottom flask closed with a silicone septum. After using a vacuum pump, nitrogen was added to obtain an anaerobic environment and then the flask was cooled in an ice-salt bath to −7 °C. A double excess solution of sodium cyclopentadienide in THF (2 M, 7.2 mL, 14.4 mmol) in 25 mL of dry tetrahydrofuran was added dropwise to the microspheres using a syringe. After 1 h, the ice-salt bath was removed, and the mixture was stirred slightly for another 24 h at ambient temperature. The reaction was quenched by pouring the reaction mixture into 100 mL of saturated NH_4_Cl solution. Subsequently, the microspheres were filtrated off and washed successively by 100 mL of acetone, ethyl alcohol-water (1/1), 3% HCl in water, water, tetrahydrofuran, and hexane. The obtained GE-Cp microspheres were dried in an oven overnight at 50 °C.

#### 2.4.2. Microspheres GE-COOH

GE-Cp microspheres (2 g, 3.0 mmol of Cp moieties) and maleic anhydride (588 mg, 6.0 mmol excess) were suspended in 50 mL of acetone in a 100 mL round bottom flask and were stirred for 2.5 h at 50 °C. The microspheres were washed thoroughly (2 × 25 mL of ethyl alcohol and 2 × 25 mL of acetone) to remove the physically absorbed anhydride moiety and dried in vacuum at ambient temperature. Then their cyclic anhydride groups were hydrolyzed using water with a few drops of sulfuric acid for 4 h at 80 °C. Under this mild condition, no ester hydrolysis occurs. The obtained GE-COOH microspheres were vacuum filtered on a Büchner funnel and washed on the filter with acetone and then oven dried overnight at 50 °C.

#### 2.4.3. Microspheres GE-BMI

To a 100 mL round-bottom flask containing 50 mL of acetone, 1 g of GE10-Cp microspheres (1.5 mmol of Cp moieties) and 1.075 g of 4,4′-bis(maleimido)diphenylmethane (3.0 mmol excess) were added. This was heated at 50 °C for 2.5 h with constant stirring.

The resulting GE-BMI microspheres were vacuum filtered on a Büchner funnel, rinsed with acetone then oven dried overnight at 50 °C.

#### 2.4.4. Microspheres GE-OH

This simple modification is based on the epoxide ring opening by the hydrolysis of GE materials with water and a few drops of sulfuric acid. The process was carried out for 3 h at 100 °C. Under such mild conditions, no ester hydrolysis takes place. The obtained GE-OH microspheres were vacuum filtered on a Büchner funnel, washed with acetone and then oven dried overnight at 50 °C.

#### 2.4.5. Microspheres GE-MAC

To a 250 mL round-bottom flask containing 100 mL of dichloromethane, 4 g of GE-OH microspheres with hydroxyl value, HV = 3.0 mmol g^−1^ (12.0 mmol) and 2.9 g of the catalyst—triethylamine (TEA, 28.8 mmol excess)—were added. This was placed in an ice bath with constant stirring. Once the temperature was about 0 °C, the dropwise step addition step of 2.5 mL methacryloyl chloride (28.8 mmol, excess) started. The process was carried out for about 1 h at a temperature not below 0 °C, then for 3 h at room temperature with constant stirring.

The resulting GE-MAC microspheres were vacuum filtered on a Büchner funnel and washed with dichloromethane and acetone and then oven dried overnight at 50 °C.

#### 2.4.6. Microspheres GE-S-COOH

First, 1.5 g of GE-MAC microspheres and 7 g of triethylamine, as catalyst, were added to a 250 mL round bottom flask containing 100 mL of dichloromethane and placed in an ice bath with constant stirring. The step of adding 2-mercaptopropionic acid in dichloromethane started dropwise (3.75 g of 2-MPA was added to a 25 mL addition funnel and made up with dichloromethane). After 1 h at a temperature of not lower than 0 °C, the process was carried out at room temperature for 3 h and next the flask was placed in a water bath for 72 h at 40 °C while stirring. The resulting GE-S-COOH microspheres were vacuum filtered on a Büchner funnel and washed with dichloromethane, methanol, tetrahydrofuran and acetone and then oven dried overnight at 50 °C.

### 2.5. Characterization

#### 2.5.1. IR Spectroscopy

The attenuated total reflectance Fourier transform infrared (FT-IR/ATR) spectra were obtained with a spectrometer with a diamond crystal (Bruker TENSOR 27, Bruker Optik GmbH, Ettlingen, Germany). The spectra were collected from 600 to 4000 cm^−1^ with 32 scans per spectrum at a resolution of 4 cm^−1^.

#### 2.5.2. Elemental Analysis

Elemental analysis of non-modified microspheres (GE), and those containing nitrogen (GE-BMI) and sulphur (GE-S-COOH), was carried out using a Perkin-Elmer analyzer (EuroEA3000, EuroVector, Pavia, Italy).

#### 2.5.3. Morphology

The morphology of the microspheres determines the shape and particle size—microsphere diameter (D_M_). These parameters were obtained from to the measurements made with the use of a particle size analyzer and micrographs taken from the scanning electron microscope (SEM).

To determine the particle size, MASTERSIZER 2000 apparatus was used (Malvern Panalytical, Malvern, United Kingdom). A laser diffraction particle size analyzer operating in the range from 0.02 to 2000 μm, which determines not only the size of the obtained microspheres but their size distribution was also applied. The sizes of the material particles were measured using an adapter for dynamic measurements with the use of an adapter in liquid dispersions. The microspheres were pre-dispersed in methanol and sonicated for 10 min. Then, using water as the dispersing phase, measurements were taken.

MORPHOLOGI G3 (Malvern Panalytical, Malvern, United Kingdom) device was also used to analyze the particle size and shape, with the possibility of measuring solid samples—as powders and in liquid dispersions.

A scanning electron microscope PHENOM from Fei Company (Thermo Fisher Scientific, Waltham, MA, USA) and high-resolution scanning microscope QUANTA 3D FEG (Fei Company, Hillsboro, OR, USA) were used.

#### 2.5.4. Porous Structure Investigations

Nitrogen adsorption–desorption measurements were made at 77 K using a volumetric adsorption analyzer ASAP 2405 (Micromeritics Inc., Norcross, GA, USA). The measurements of the surface properties of the copolymers were undertaken by outgassing the samples at 140 °C for 2 h. The specific surface areas were calculated by the Brunauer–Emmet–Teller (BET) method for the adsorption data in the range of a relative pressure p/p_0_ 0.05 to 0.25. The total pore volume was estimated from a single point adsorption at a relative pressure of 0.985. The pore size distributions (PSD) were obtained from the desorption branch of the isotherm using the Barrett–Joyner–Halenda (BJH) procedure. Due to the small range of pore size detection, nitrogen adsorption porometry is mainly used for mesopore characterization [39,40].

In a swollen state, the copolymers were characterized by an inverse size exclusion chromatography (ISEC). The main assumption in this method is that in the good solvent chains of polystyrene macromolecules form coils of a diameter corresponding to the polymer molecular weights. The diameter of the probe molecules (Φ [Å]; 1 Å = 0.1 nm) was calculated from Equation (1):

(1) $$\Phi =0.62M_{W}{}^{0.59}$$

where M_w_ is the gram-molecular weight of the probe.

The diameter of the probe molecule is associated with a pore diameter (Φ), which corresponds to the smallest pore allowing an unhindered access for the probe of a given molecular weight. As the pore-size probes, toluene, alkylphenones, phthalates, and polystyrene standards were used. The cumulative pore size distribution was determined from the plot 1−K_0_(EC) versus logΦ, where K_0_(EC) is the distribution constant in exclusion chromatography, calculated from Equation (2):

(2) $$K_{0}\left(\mathrm{EC}\right)=\frac{V_{R}-V_{0}}{V_{p}}=\frac{V_{R}-V_{0}}{V_{i}-V_{0}}$$

where

V_R_—the retention volume of the probe
V_0_—the interstitial volume equal to the retention volume of a totally excluded molecule
V_i_—the retention volume of a totally included molecule
V_p_—the pore volume.

As previously, V_i_ is equal to the retention volume of toluene [41]. Retention volumes of toluene, alkylphenones, phthalates, and polystyrene standards were determined with a Hewlett-Packard HP-1050 liquid chromatograph equipped with a diode-array UV detector, a Rheodyne 7125 injection valve with the 20-μL sample loop and columns (100 mm × 4.1 mm I.D. (Internal Diameter)) packed with the copolymers described above. In the exclusion chromatography measurements, tetrahydrofuran (THF) was the mobile phase at the flow rate 1.0 mL min^−1^. Each substance was injected separately as a 0.1% solution in THF.

#### 2.5.5. HPLC Separations

The obtained microspheres were slurry packed into the 100 mm × 4.1 mm i.d. stainless HPLC column using Pack in a Boxsystem (SSI, State College, PA, USA) under the pressure of 20 MPa according to the procedure described previously [42]. Chromatographic separations were performed using the liquid chromatograph (UltiMate 3000, Thermo Scientific Dionex, Sunnyvale, CA, USA) equipped with a UV-VIS detector with the DAD matrix. Using the ACN-phosphate buffer (pH = 7) and MeOH-phosphate buffer (pH = 7) of different organic solvent concentrations as mobile phases, the following were separated: alkylbenzenes, alkyl aryl ketones, alkyl benzoates, *N*-alkylanilines, and alkyl aryl ethers. The flow rate of mobile phases was 1 mL min^−1^. Chromatograms were recorded using the UV detector operating at 254 nm.

## 3. Results and Discussion

In one-step swelling and polymerization of poly(GMA-co-EGDMA) beads, the morphology and porous structure can be controlled not only by the amount of seed and crosslinker, but also by the type, concentration, and cosolvency of porogen organic solvents.

### 3.1. Polystyrene Seeds

The particle size distribution (PSD) was determined with the MASTERSIZER 2000 apparatus using the laser diffraction method. It can be seen that the obtained PS microspheres have a narrow particle size distribution with an average particle diameter (d0.5) of 1.91 ± 0.05 µm.

**Figure 1:**
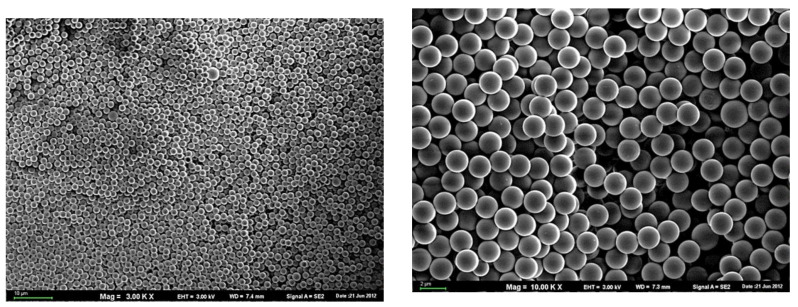
SEM micrographs of PS seeds.

On the basis of the SEM micrographs (Figure 1), it can be also observed that the monodisperse PS microspheres are characterized by good sphericality and surface smoothness, which may indicate a lack of porous structure.

### 3.2. Porous Microspheres GE

**Figure 2:**
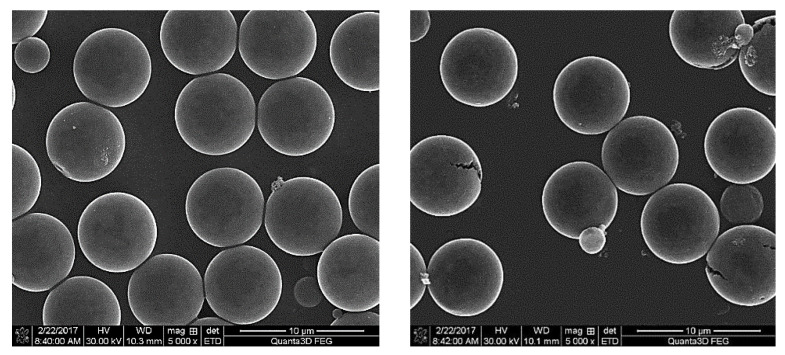
SEM micrographs of GE microspheres.

Based on the SEM micrographs (Figure 2), it can be seen that the GE microspheres obtained by seed swelling polymerization are spherical and monodisperse. Their diameter is much larger compared with the starting seeds. The surface of the obtained material is not smooth, which may indicate the presence of a porous structure. Large gaps, crevices and cracks are observed.

**Figure 3:**
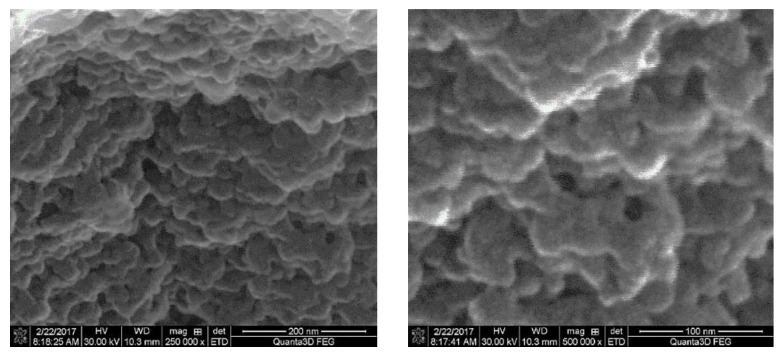
SEM micrographs of the surface of GE microsphere.

Based on the SEM micrographs (Figure 3), one can see that the surface of the GE microspheres is not smooth, but rough and irregular, similar to that of the cauliflower. Irregularities confirm the presence of pore channels, and thus the porous structure of the material. This study also made it possible to confirm the structure of a single microsphere made of nuclei.

#### 3.2.1. Effect of EGDMA as a Crosslinker

According to Table 1, the amount of ethylene glycol dimethacrylate as a crosslinker affects morphology and porous structure. Due to the structures of glycidyl methacrylate and ethylene glycol dimethacrylate, which are almost the same, the miscibility of these two monomers is high.

The specific surface area and pore volume increase with increasing amounts of EGDMA from 43 to 132 m^2^ g^−1^ and from 0.19 to 0.43 cm^3^ g^−1^, respectively. The GE2 material, which is the most crosslinked (molar ratio—1:1.40), has the highest values compared to the GE1 (1:0.70) and GE3 (1:0.35) materials. Through the polymerization process, the most developed porous structure is obtained, while simultaneously forming the microspheres with the smallest diameter. One can observe that with decreasing EGDMA, the diameters of microspheres increase from 8.85 to 9.52 µm.

The influence of the crosslinking monomer on the porous structure and morphology of the obtained materials can be explained as follows. With a higher content of EGDMA, the degree of crosslinking of the primary microspheres was greater, and their ability to dissolve each other decreased. As a result of phase separation, the internal structure of the microspheres was built of evenly distributed, tightly packed small spheres formed during the binding and agglomeration of microspheres. The voids formed between them were interconnected, creating pores in the range of micro and mesopores. On the other hand, with a low crosslinking monomer content, the phase separation was difficult, and the weakly crosslinked microspheres mutually dissolved, being able to absorb large amounts of solvent, and formed large agglomerates with larger pores between them as a result.

In the further studies, GE2 was used as the reference material.

#### 3.2.2. Effect of Seed Amount

From Table 2, one can see that the swelling of different amounts of PS seeds with the same amount of monomers mixture and pore-forming diluent causes differences in the resulting porous structure (specific surface area, pore volume) and morphology. The microspheres with the largest specific surface area, volume and pore diameter wree the GE4 material synthesized with the smallest amount of seeds (0.25 g). This material is also characterized by the largest diameter (11.12 µm) among the obtained particles.

#### 3.2.3. Effect of Porogen Concentration

According to Table 3, the use of toluene as a pore-forming diluent influences the morphology and porous structure of the obtained polymeric microspheres.

When the amount of toluene increases, the phase separation is slower, and smaller macrogel agglomerates are formed. Consequently, the porosity of the microspheres increases. In Table 3, an increase in the specific surface area from 10 to 178 m^2^ g^−1^, the pore volume from 0.08 to 0.52 cm^3^ g^−1^, and the diameter of the microspheres from 8.53 to 10.36 µm can be observed. On the other hand, there is a decrease in the diameter of the formed pores from 29.5 to 11.7 nm, which can chiefly be explained by the fact that toluene, as a good solvent for the precipitating polymer, causes the formation of smaller pores in the structure.

Even the lack of a pore-forming diluent in the swelling step causes formation of some pores (material GE8). This can be explained by the fact that the moderate miscibility of the monomers in water has a long-term affinity for the aqueous phase, and the formation of pores with a small volume (0.08 cm^3^ g^−1^) and specific surface area (10 m^2^ g^−1^) is possible. This material possesses pores with much larger diameters (29.5 nm).

#### 3.2.4. Effect of Porogen Type

In Table 4, the effects of the type of porogenic solvent on the morphology and porous structure are shown. Five solvents with different nature of intermolecular interactions were considered. It was observed that for the GMA:EGDMA (molar ratio—1:1.4), the solvating (SOL) solvents were toluene, cyclohexanol and chlorobenzene, whereas 1-decanol and dibutyl phthalate were non-solvating (NONSOL).

The GE10 microspheres are characterized by the largest specific surface area (161 m^2^ g^−1^), pore volume (0.55 cm^3^ g^−1^), and pore diameter (13.6 nm). They were obtained using chlorobenzene as the porogen. This material forms particles with the largest diameter (10.08 µm).

The material obtained with the use of cyclohexanol (GE9) was also characterized by large values of specific surface area and pore volume, but this product was not obtained in the form of monodisperse particles. A similar polydisperse material with large mean pore diameter (27.5 nm) is GE11. This was obtained using dibutyl phthalate as the pore-forming solvent.

The use of 1-decanol instead of toluene (GE12) gave a material that was not in the form of microspheres.

#### 3.2.5. Effect of 1-Decanol as a Porogen Cosolvent

A mixture of toluene and 1-decanol as a co-solvent in various proportions was used in the synthesis of microspheres. The effects of the co-solubility of organic pore-forming solvents on the morphology and porous structure are presented in Table 5.

Even small additions of alcohol cause an increase of specific surface area and pore volume compared with the GE2 material. With up to 50% of 1-decanol as a cosolvent, the surface area and the pore volume increased significantly (materials GE14 and GE13), whereas for the materials with the largest concentrations (GE15 and GE12), these values decreased. Additionally, products were not obtained in the monodisperse form.

#### 3.2.6. Effect of Surface Modification

In the modification reactions, GE10 polymer was used as the starting material. As shown in Table 6, surface modification causes changes in the porous structure, reducing the specific surface area and pore volume. The largest observed change concerned the GE-S-COOH material, where S_BET_ was 97 m^2^ g^−1^ and V_p_ = 0.398 cm^3^ g^−1^. This material also had the largest pore diameter (16.4 nm). For the remaining materials, the changes were minor.

Due to the fact that water molecules change the chemical nature of the GE-OH copolymer, this leads to its shrinkage. The slightly greater loosening effect of the net is visible for the polymer with the carboxyl groups (GE-COOH). The narrowing of the pores of the GE-BMI and GE-COOH materials can be explained by the presence of large bismaleimide and norbornene dicarboxylic fragments in the pores.

In both cases, some pores were no longer accessible to nitrogen molecules.

### 3.3. Spectroscopic Analysis of Functionalized Stationary Phases

The spectroscopic analysis of the starting GE material and modified GE-COOH have been discussed in detail previously [43].

**Figure 4:**
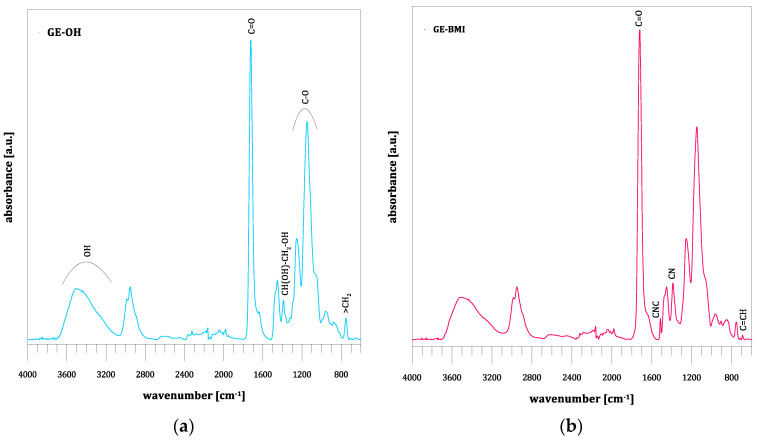

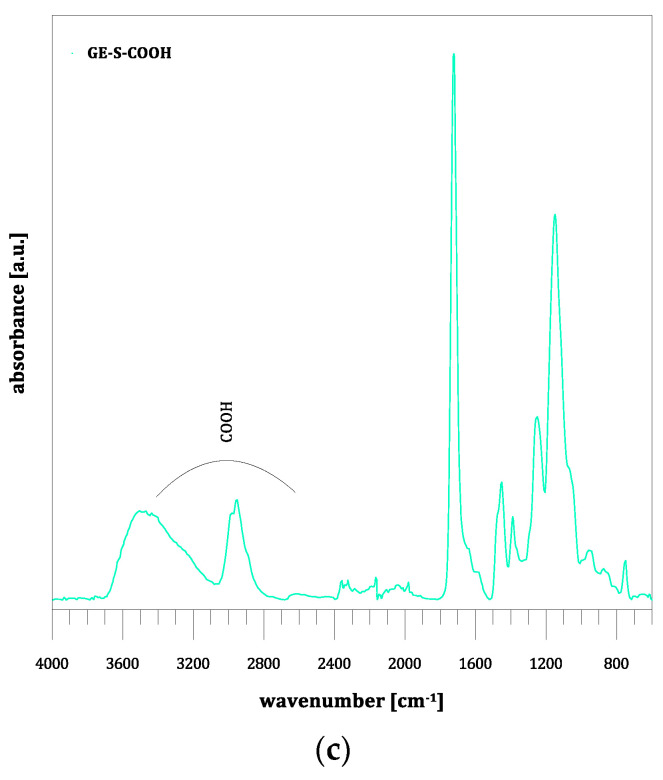
ATR/FT-IR spectrum of the obtained porous microspheres: (**a**) GE-OH; (**b**) GE-BMI; (**c**) GE-S-COOH.

In the FT-IR spectra (Figure 4a–c), a high-intensity band at 1727 cm^−1^, corresponding to the vibrations of the C=O carbonyl group of aliphatic methacrylic esters, is visible. The next three absorption bands, at 1259, 1151 and 1060 cm^−1^, characteristic of methacrylic esters, come from the C-O vibrations. There are no bands at 905 and 846 cm^−1^ corresponding to the asymmetrical and symmetrical C-O-C deformation vibrations of the epoxy ring. This makes it possible to confirm the modification step of the GMA-derived fragment in the polymer network. The band at 754 cm^−1^ corresponding to the methylene groups >CH_2_ confirms the presence of the EGDMA-derived fragment being incorporated into the polymer chain.

Figure 4a shows the spectrum of hydrolyzed GE microspheres. The bands at 905 cm^−1^ and 846 cm^−1^ characteristic of the epoxy ring disappear, which indicates that this group was completely converted into a hydroxyl one. In the spectrum, a wide band at 3800 to 3200 cm^−1^ corresponding to the vibrations of OH groups is visible. The increase in the intensity of the bands at 1430 to 1330 cm^−1^ can be attributed to the vibrations coming from the resulting fragment of the vic-diol -CH(OH)-CH_2_OH.

The addition of 4,4′-(bismaleimido)diphenylmethane to the microsphere structure can be observed in the ATR/FTIR spectrum of GE-BMI microspheres (Figure 4b). The appearance of stretching imide bands: I (1720 cm^−1^, C=O), II (1513 cm^−1^, CNC), III (1393 cm^−1^, CN), and IV (695 cm^−1^, C=CH) suggests that the Diels-Alder click reaction proceeds on the surface of the porous polymeric microspheres.

The assumption that during the reaction, the maleimide group is transformed into its saturated analogue—succinimide—can be confirmed by the disappearance of the bending bands characteristic of maleimide at values of about 690 cm^−1^ (ring bending) and 830 cm^−1^ (out-of-plane bending). The low intensity of these bands may suggest that there is also a fragment of unreacted maleimide groups at the other end of the 4,4′-(bismaleimido)diphenylmethane. It is difficult to accurately and unambiguously confirm the presence of the C-N-C group from maleimide or succinimide which give bands at 1150 and 1180 cm^−1^ because their overlapping takes place.

In Figure 4c, showing the FTIR spectrum of GE-S-COOH microspheres, the absorption band at 2580 cm^−1^ derived from the thiol stretching vibrations from 2-mercaptopropionic acid (2-MPA) is significant. This peak is not observed, which can be considered to correspond to covalent bond formation between the SH and C=C groups present on the surface of the microspheres. There was also a decrease in the intensity of the absorption bands at about 1630 cm^−1^ and 950 cm^−1^, characteristic of the vinyl groups. The appearance of a wide band ranging from 3400 cm^−1^ to 2400 cm^−1^ confirms the addition and presence of a characteristic carboxyl group derived from 2-MPA.

### 3.4. Elemental Analysis

To confirm the Diels-Alder reaction with 4,4′-(bismaleimido)diphenylmethane and the thio-Michael addition reaction with 2-mercaptopropionic acid on the surface of the polymeric microspheres, an elemental analysis was performed. The percentages of carbon, hydrogen, nitrogen and sulfur (CHNS) for the non-modified and functionalized polymeric materials are presented in Table 7.

The non-modified microspheres (GE10) consisting aliphatic chains of glycidyl methacrylate and ethylene glycol dimethacrylate contained carbon (58.54%), oxygen (34.24%) and hydrogen (7.22%), respectively.

After chemical modification with 4,4′-bis(maleimido)diphenylmethane, the obtained GE-BMI material was characterized by an increase in the percentage of nitrogen (1.19%). Its presence confirms the surface functionalization. The increase of the oxygen percentage was a result of the bismaleimide molecule.

A similar case can be observed for the GE-S-COOH material. After chemical modification with 2-mercaptopropionic acid, the sulfur concentration in the obtained GE-S-COOH material was equal to 0.44%. An increase in oxygen concentration (41.10%) can also be found. This indicates the incorporation of methacrylate chains derived from methacryloyl chloride and 2-mercaptopropionic acid in the polymer surface modification stages.

### 3.5. Porous Structure Investigations

The porous structures of the synthesized microspheres were characterized using both nitrogen adsorption/desorption measurements and inverse exclusion chromatography. The former technique refers to the polymeric material in the dry state, while the latter refers to the polymer that is wetted by good solvent. In a swollen state, the porous structure of the polymer was similar to that under liquid chromatography conditions. From this point of view, ISEC is more informative for the characterization of HPLC stationary phases [14]. In this technique, the differences in the retention volumes of molecules with the diameters smaller than 20 Å and dinonyl phthalate (Φ = 22 Å) determine the volume occupied by micropores in the internal structure of the polymers. The total pore volume is expressed by the difference between the retention of the completely excluded molecule and the compound penetrating the polymer network (toluene, Φ = 9.0 Å).

The results of nitrogen adsorption measurements and ISEC are presented in Table 8.

**Figure 5:**
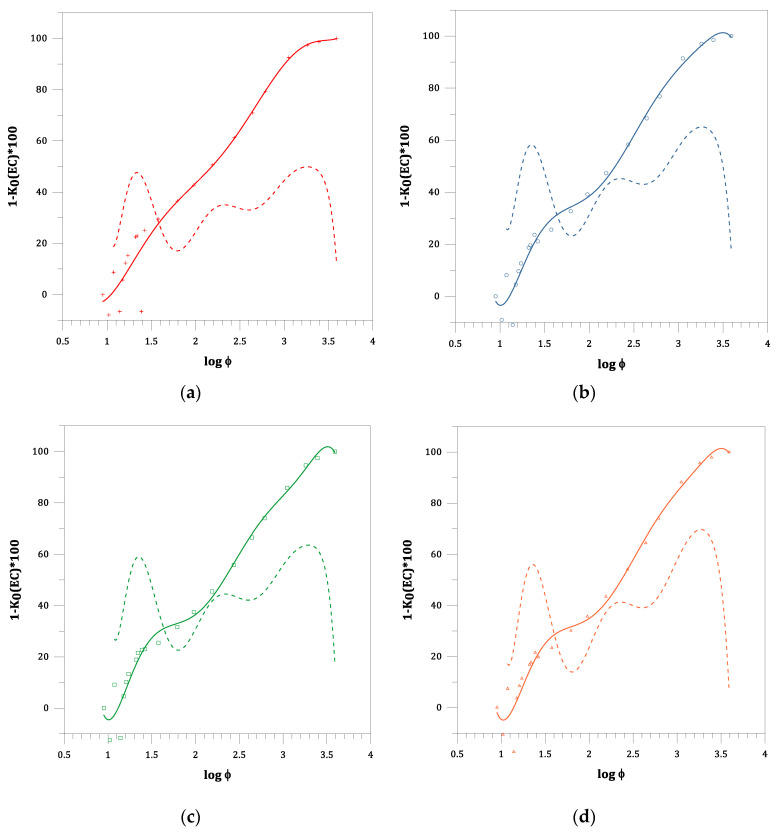
ISEC cumulative (―) and differential (‒ ‒) pore size distribution curves for the obtained copolymers: (**a**) GE; (**b**) GE-OH; (**c**) GE-COOH; (**d**) GE-S-COOH.

The results in Table 8 show that all synthesized microspheres have a developed porous structure. The pore volumes measured by the nitrogen adsorption method are generally smaller than those determined by ISEC. Moreover, this technique does not confirm the existence of micropores in the structure of any of the studied materials. On the other hand, the results of the ISEC indicate the existence of micropores in the internal structure of microspheres. Their contribution for the starting material exceeds 20% and slightly decreases after modification (Figure 5).

The obtained data confirm the thesis that the polymers used as stationary phases, regardless of the fact that they show high mechanical resistance, reveal undesirable microporosity when in contact with strong organic solvents. As a result, they can swell slightly. This problem is less important when eluents with a high water content are used, because under these conditions, the microporous structure is closed or unavailable. In this case, only meso- and macroporores are open for the solutes [44].

While each standard has a known molecular weight, an ISEC curve representing the relationship between logM_w_ and the retention volume can be plotted. If the M_w_ is further correlated with the diameter of the polystyrene standards, the pore size distribution determined by the column can be described.

### 3.6. Chromatographic Separations

To evaluate the influence of the chemical structure of the stationary phase on retention, the method reported by Smith was used [45]. He introduced three mobile phases containing a phosphate buffer of pH = 7 with the following compositions: methanol-buffer (90:10), ACN-buffer (70:30) and tetrahydrofuran-buffer (40:60). These phases have a similar elution strength despite the fact that each of them contains a solvent with a significantly different character in terms of intermolecular interactions. In their presence, the separation of alky aryl ketones on the nonpolar styrene-divinylbenzene column was obtained in a time not exceeding 15 min.

**Figure 6:**
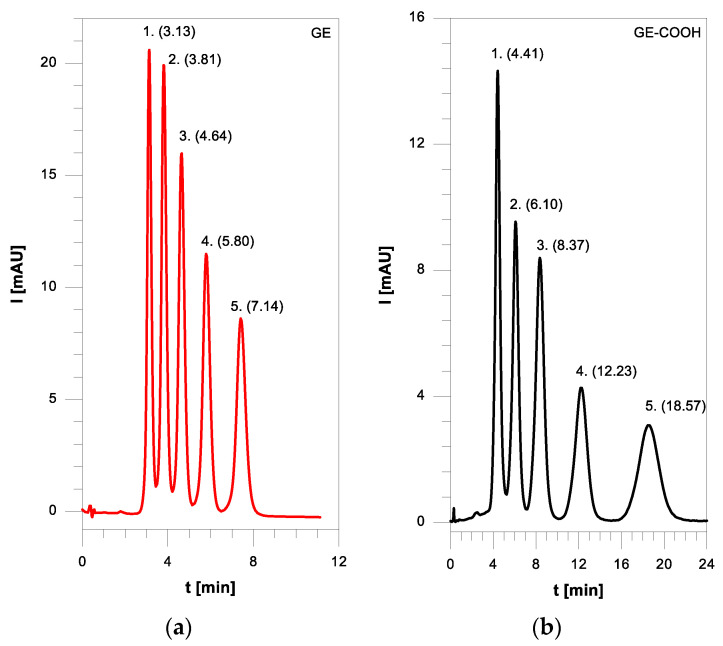
Separation of the mixture of homologous alkyl benzenes, ACN-buffer: (**a**) GE; (**b**) GE-COOH.

This method was adopted to evaluate the properties of more polar polymeric stationary phases [46,47]. Our current studies show that separation of the test mixtures on the obtained polar phases is possible with a much greater content of mobile phases than water phase. Short retention time and satisfactory separation were obtained on the starting GE stationary phase when the composition of the mobile phase was changed to contain 35% ACN or 40% methanol (Figure 6).

**Figure 7:**
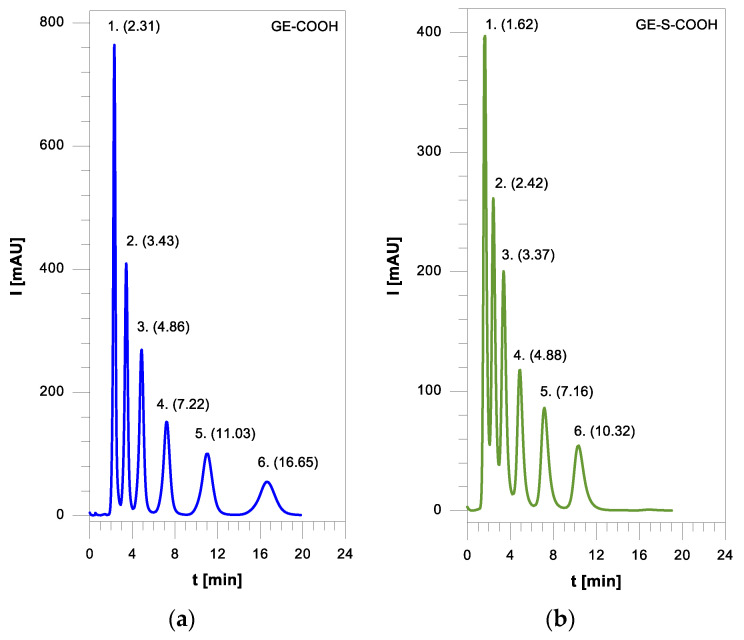
Separation of the mixture of homologous alkyl aryl ketones, ACN-buffer (35:65, v:v): (**a**) GE-COOH; (**b**) GE-S-COOH.

**Figure 8:**
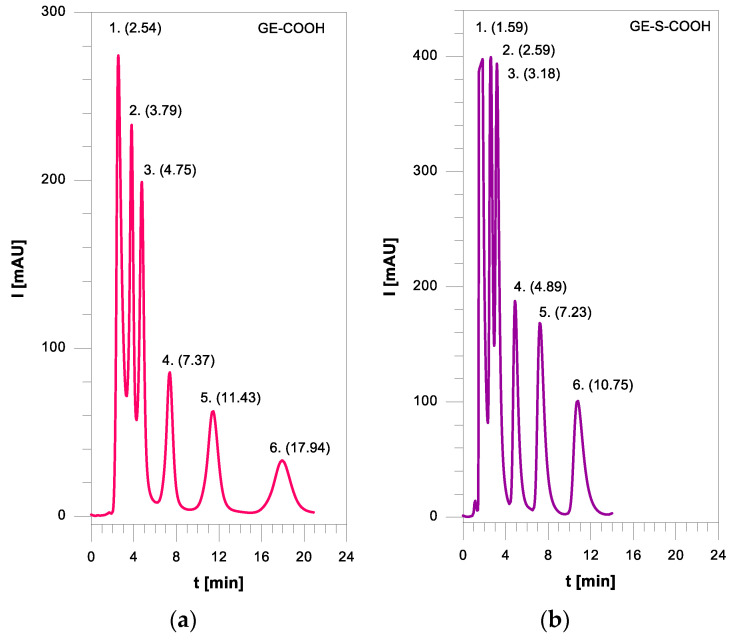
Separation of the mixture of homologous *N*-anilines, ACN-buffer (35:65, v:v): (**a**) GE-COOH; (**b**) GE-S-COOH.

**Figure 9:**
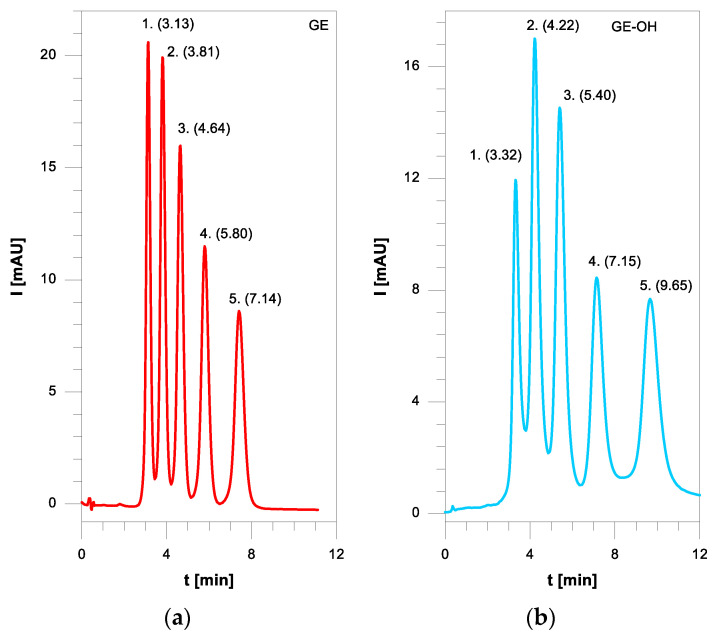
Separation of the mixture of homologous alkyl benzenes, ACN-buffer: (**a**) GE; (**b**) GE-OH.

Separations of mixtures of the tested compounds on the functionalized phases were performed with the same mobile phase compositions. The exemplary separations are shown in Figure 7, Figure 8 and Figure 9. The separations of homologous alkyl aryl ketones on the polymeric phases with the carboxylic groups (GE-COOOH and GE-S-COOOH) are presented in Figure 7, whereas those of the mixture of polar *N*-anilines in Figure 8. Although the separation of anilines was incomplete for the initial components of the mixture, the peaks were symmetrical. On the phase with sulfur, the retention times of *N*-anilines were shorter. Nonpolar alkylbenzenes were well separated on both the starting GE stationary phase and that with the hydroxyl functional groups (Figure 9).

Porous polymeric microspheres characterized by carboxyl groups functionalized surfaces can find application in methods other than HPLC chromatography. They can be used as sorbents in the extraction of SPE for the preconcentration of toxic aromatic compounds from water samples, as well as chelating ion exchangers.

## 4. Conclusions

The porous poly(GMA-co-EGDMA) microspheres were synthesized by one-step swelling and free-radical polymerization. In this method, the morphology and porous structure can be controlled not only through the amount of seed and crosslinker, but also through the type, concentration, and cosolvency of porogen organic solvents.

The oxirane groups in the material structure are highly reactive and undergo ring-opening reactions where hydrolysis allows the introduction of a diol moiety. The conjugated reactions firstly with sodium cyclopentadienide and then the Diels-Alder with maleic anhydride result in a carboxylic acid part being obtained. The analogous DA click reaction with 4,4′-bis(maleimido)diphenylmethane made it possible to introduce maleimide groups on the surface. A vic-diol fragment is an ideal starting point for modification due to the reaction with methacryloyl chloride. The vinyl bonds introduced in this way were then reacted with 2-mercaptopropionic acid by the thiol-Michael addition click reaction, through which carboxylic acid and thioether groups are obtained.

The modification reaction steps were confirmed by infrared spectroscopy. The results were also extended to the elemental analysis. This confirms the incorporation of nitrogen and sulfur after functionalization, and thus their presence in the polymeric material.

Microspheres possessing epoxide, diol, and carboxylic acid functional groups are very attractive. Novel stationary phases are prospective packing materials for high-performance liquid chromatography. Smaller consumption of the organic solvent as a mobile phase in HPLC has a significant impact on the fact that the use of functional porous polymeric microspheres is part of the trend of green chromatography.

## Figures and Tables

**Table 1:** Effect of crosslinker on morphology and porous structure.

Sample	G:E [a]	S_BET_ [b]	V_p_ [c]	D_p_ [d]	Morphology	D_M_ [e]
GE2	1:1.40	131.69 ± 0.59	0.43	13.1	Monodisp.	8.85 ± 0.13
GE1	1:0.70	71.16 ± 0.34	0.31	17.6	Monodisp.	9.28 ± 0.14
GE3	1:0.35	42.76 ± 0.22	0.19	17.5	Monodisp.	9.52 ± 0.14

[a] GMA:EGDMA molar ratio. [b] Specific surface area, m^2^ g^−1^. [c] Pore volume, cm^3^ g^−1^. [d] Pore diameter, nm. [e] Microsphere diameter, μm.

**Table 2:** Effect of PS seed amount on morphology and porous structure.

Sample	PS [a]	S_BET_ [b]	V_p_ [c]	D_p_ [d]	Morphology	D_M_ [e]
GE4	0.25	166.59 ± 0.61	0.57	13.7	Monodisp.	11.12 ± 0.17
GE2	0.45	131.69 ± 0.59	0.43	13.1	Monodisp.	8.85 ± 0.13
GE5	0.65	129.42 ± 0.60	0.37	11.5	Monodisp.	8.30 ± 0.13

[a] Seed amount, g. [b] Specific surface area, m^2^ g^−1^. [c] Pore volume, cm^3^ g^−1^. [d] Pore diameter, nm. [e] Microsphere diameter, μm.

**Table 3:** Effect of porogen amount on morphology and porous structure.

Sample	Toluene [a]	S_BET_ [b]	V_p_ [c]	D_p_ [d]	Morphology	D_M_ [e]
GE8	0	10.43 ± 0.08	0.08	29.5	Monodisp.	8.53 ± 0.13
GE6	11.6	122.00 ± 0.45	0.38	12.5	Monodisp.	8.68 ± 0.13
GE2	16.6	131.69 ± 0.59	0.43	13.1	Monodisp.	8.85 ± 0.13
GE7	21.6	177.71 ± 0.77	0.52	11.7	Monodisp.	10.36 ± 0.16

[a] Porogen amount, mL. [b] Specific surface area, m^2^ g^−1^. [c] Pore volume, cm^3^ g^−1^. [d] Pore diameter, nm. [e] Microsphere diameter, μm.

**Table 4:** Effect of porogenic solvent type on morphology and porous structure.

Sample	Solvent [a]	S_BET_ [b]	V_p_ [c]	D_p_ [d]	Morphology	D_M_ [e]
GE2	Toluene	131.69 ± 0.59	0.43	13.1	Monodisp.	8.85 ± 0.13
GE9	Cyclohexanol	158.67 ± 0.62	0.56	14.0	Polydisp.	8.73 ± 0.23
GE10	Chlorobenzene	161.32 ± 0.62	0.55	13.6	Monodisp.	10.08 ± 0.15
GE11	Dibutyl phthalate	49.10 ± 0.14	0.34	27.5	Polydisp.	9.71 ± 0.35
GE12	1-decanol	31.04 ± 0.11	0.05	6.9	Non-sperical	-

[a] Porogen solvent. [b] Specific surface area, m^2^ g^−1^. [c] Pore volume, cm^3^ g^−1^. [d] Pore diameter, nm. [e] Microsphere diameter, μm.

**Table 5:** Effect of cosolvency on morphology and porous structure.

Sample	1-Decanol [a]	S_BET_ [b]	V_p_ [c]	D_p_ [d]	Morphology	D_M_ [e]
GE2	0	131.69 ± 0.59	0.43	13.1	Monodisp.	8.85 ± 0.13
GE14	25	179.48 ± 0.77	0.56	12.5	Polydisp.	11.86 ± 0.21
GE13	50	167.11 ± 0.65	0.59	14.2	Polydisp.	9.57 ± 0.25
GE15	75	53.70 ± 0.30	0.18	13.4	Polydisp.	9.45 ± 0.34
GE12	100	31.04 ± 0.11	0.05	6.9	Non-sperical	-

[a] Amount in porogen solvent mixture, %. [b] Specific surface area, m^2^ g^−1^. [c] Pore volume, cm^3^ g^−1^. [d] Pore diameter, nm. [e] Microsphere diameter, μm.

**Table 6:** Effect of surface modification on porous structure.

Sample	Modification [a]	S_BET_ [b]	V_p_ [c]	D_p_ [d]
GE10	Epoxy, non-modified	161.32 ± 0.62	0.55	13.6
GE-OH	Vic-Diol	133.53 ± 0.51	0.47	14.2
GE-S-COOH	Thioether-carboxyl	96.97 ± 0.16	0.40	16.4
GE-COOH	Dicarboxyl	138.24 ± 0.78	0.50	14.5
GE-BMI	Bismaleimide	142.32 ± 0.64	0.44	12.5

[a] Functional groups on the surface. [b] Specific surface area, m^2^ g^−1^. [c] Pore volume, cm^3^ g^−1^. [d] Pore diameter, nm.

**Table 7:** The results of the CHNS elemental analysis of polymeric microspheres.

Sample	%C	%H	%N	%S	%O [a]
GE	58.54	7.22	0.0	0.0	34.24
GE-BMI	55.69	6.82	1.19	0.0	36.30
GE-S-COOH	52.08	6.38	0.0	0.44	41.10

[a] %O = 100% − (%C + %H + %N + %S).

**Table 8:** Porous structure of chemically modified copolymers.

Porous Structure	GE		GE-OH		GE-COOH		GE-S-COOH	
	BET [d]	ISEC [e]	BET [d]	ISEC [e]	BET [d]	ISEC [e]	BET [d]	ISEC [e]
S [a]	161		134		131		97	
V_p_ [b]	0.55	0.70	0.47	0.64	0.50	0.58	0.40	0.52
V_M_ [c]	0.0	0.16	0.0	0.15	0.0	0.12	0.0	0.12

[a] Specific surface area, m^2^ g^−1^. [b] Pore volume, mL g^−1^. [c] Volume of micropores, mL g^−1^. [d] Nitrogen adsorption measurements. [e] Inverse size-exclusion chromatography.

## Data Availability

Data sharing is not applicable to this article.
